# Nontuberculous Mycobacterial Disease in Children – Epidemiology, Diagnosis & Management at a Tertiary Center

**DOI:** 10.1371/journal.pone.0147513

**Published:** 2016-01-26

**Authors:** Marc Tebruegge, Anastasia Pantazidou, Duncan MacGregor, Gena Gonis, David Leslie, Luigi Sedda, Nicole Ritz, Tom Connell, Nigel Curtis

**Affiliations:** 1 Department of Paediatrics, The University of Melbourne, Parkville, Victoria, Australia; 2 Infectious Diseases Unit, Royal Children’s Hospital Melbourne, Parkville, Victoria, Australia; 3 Murdoch Children’s Research Institute, Parkville, Victoria, Australia; 4 Academic Unit of Clinical & Experimental Sciences, Faculty of Medicine, University of Southampton, Southampton, United Kingdom; 5 NIHR Respiratory Biomedical Research Unit, University Hospital Southampton NHS Foundation Trust, Southampton, United Kingdom; 6 Department of Anatomical Pathology, Royal Children’s Hospital Melbourne, Parkville, Victoria, Australia; 7 Department of Microbiology, Royal Children’s Hospital Melbourne, Parkville, Victoria, Australia; 8 Mycobacterium Reference Laboratory, Victorian Infectious Diseases Reference Laboratory, North Melbourne, Australia; 9 Department of Geography and Environment, University of Southampton, Southampton, United Kingdom; 10 Infectious Diseases Unit, University Children’s Hospital Basel, Basel, Switzerland; National Institute of Infectious Diseases, JAPAN

## Abstract

**Background:**

There are limited data on the epidemiology, diagnosis and optimal management of nontuberculous mycobacterial (NTM) disease in children.

**Methods:**

Retrospective cohort study of NTM cases over a 10-year-period at a tertiary referral hospital in Australia.

**Results:**

A total of 140 children with NTM disease, including 107 with lymphadenitis and 25 with skin and soft tissue infections (SSTIs), were identified. The estimated incidence of NTM disease was 0.6–1.6 cases / 100,000 children / year; no increasing trend was observed over the study period. Temporal analyses revealed a seasonal incidence cycle around 12 months, with peaks in late winter/spring and troughs in autumn. Mycobacterium-avium-complex accounted for most cases (77.8%), followed by *Mycobacterium ulcerans* (14.4%) and *Mycobacterium marinum* (3.3%). Polymerase chain reaction testing had higher sensitivity than culture and microscopy for acid-fast bacilli (92.0%, 67.2% and 35.7%, respectively). The majority of lymphadenitis cases underwent surgical excision (97.2%); multiple recurrences in this group were less common in cases treated with clarithromycin and rifampicin compared with clarithromycin alone or no anti-mycobacterial drugs (0% versus 7.1%; OR:0.73). SSTI recurrences were also less common in cases treated with two anti-mycobacterial drugs compared with one or none (10.5% versus 33.3%; OR:0.23).

**Conclusions:**

There was seasonal variation in the incidence of NTM disease, analogous to recently published observations in tuberculosis, which have been linked to seasonal variation in vitamin D. Our finding that anti-mycobacterial combination therapy was associated with a reduced risk of recurrences in patients with NTM lymphadenitis or SSTI requires further confirmation in prospective trials.

## Introduction

Currently there are more than 140 recognized species of nontuberculous mycobacteria (NTM).[1] NTM can cause a broad range of infections in humans, including lymphadenitis, skin and soft tissue infections (SSTIs), pulmonary disease, otitis media, and osteomyelitis [1]. The overall incidence of NTM disease increased significantly in the 1980s and 1990s, in parallel with the rise of AIDS cases [1,2]. Data suggest the incidence of NTM disease in immunocompetent individuals has also risen over recent decades, although it remains unclear whether this represents a true increase or reflects increased awareness and/or improvements in diagnostic methods [1,3,4].

Despite the description of NTM disease in humans in the 1930s [5], there remains a surprising paucity of data in children. The vast majority of previous reports are case series or studies that included fewer than 50 cases, which frequently lacked detail on the diagnostic workup, management (especially anti-mycobacterial treatment) and outcome [1]. Consequently, there remains considerable uncertainty about the optimal management of NTM disease in children, as highlighted in the latest American Thoracic Society (ATS) / Infectious Diseases Society of America (IDSA) statement [6].

This study aimed to determine the epidemiology, spectrum of disease, accuracy of diagnostic methods, treatment and outcome in a large cohort of children with NTM disease at the largest pediatric tertiary referral hospital in Australia.

## Patients and Methods

### Case definitions

NTM lymphadenitis and SSTIs were defined as i) isolation of NTM in culture, ii) detection of NTM by polymerase chain reaction (PCR), or iii) presence of acid-fast bacilli in conjunction with caseating or non-caseating granulomata on histological examination of the relevant tissue (ie lymph node aspirate/tissue sample or skin/soft tissue sample, respectively) in the absence of risk factors for tuberculosis (TB). Pulmonary NTM disease was defined according to the latest ATS/IDSA statement on NTM infections [6]. Other forms of NTM disease were defined as isolation of NTM in culture or detection by PCR in a sample from a normally sterile site or body fluid.

### Patients and data collection

Potential cases (aged 0–18 years) between September 2000 and September 2010 were identified from the databases of the Histopathology and the Microbiology Departments of the Royal Children’s Hospital Melbourne (RCH), and from the database of the Victorian Infectious Diseases Reference Laboratory (VIDRL). The former was searched for reports that included the keyword ‘granuloma*’; the latter two for positive mycobacterial culture and PCR results. The RCH provides healthcare for a population of approximately 1.19 million children and adolescents in the state of Victoria, Australia based on official 2006 census data [7]. Data were extracted from laboratory and medical records into standardized data collection sheets.

### Laboratory methods

Samples were routinely processed at the RCH Microbiology Department, which performed mycobacterial cultures and referred isolates to VIDRL for identification, which was done by use of phenotypic methods, or 16S rRNA gene or internal transcribed spacer region sequencing if the former produced no definitive result. PCRs were also performed at VIDRL, using a real-time TaqMan (Roche Molecular Diagnostics, Castle Hill, Australia) PCR for *Mycobacterium ulcerans* that targets a multi-copy insertion sequence, as previously described [8]; other NTM were identified by a generic mycobacterial PCR that amplifies the single copy 16S-23S intergenic spacer region, followed by species determination by restriction fragment length polymorphism or sequencing, as previously described [9]. QuantiFERON-TB Gold (QFT) assays were performed at VIDRL according to manufacturer’s instructions.

### Statistical analysis

The data were analyzed with STATA (StataCorp; College Station, TX, US) and Prism (GraphPad; La Jolla, CA, US). For comparisons of two groups, Mann Whitney *U* and two-tailed Fisher’s exact tests were used for the analysis of continuous and categorical data, respectively. For comparisons of multiple groups, Kruskal Wallis and chi-square tests were used, as appropriate. Incidence estimates were based on official census data using the child and adolescent population figure from 2006 (1.19 million) as the denominator. Disease seasonality was analyzed using discrete and continuous wavelet transform methods built into R software (Free Software Foundation; Boston, MA, US), following detrending and denoising of the data [10]. Trend was removed by subtracting the monthly estimated mean obtained from a trend regression (where the independent variable was the number of cases/month, and the dependent variable was the time in months). Denoising was performed using a discrete wavelet transform with Daubechies length 6 wavelet filter.

### Ethical approval

This study was approved by the Royal Children’s Hospital Melbourne Human Research Ethics Committee (HREC Approval No. 31053). The HREC did not require consent to be obtained from patients/carers due to the retrospective nature of the research project. All data were anonymized and de-identified prior to analysis.

## Results

A total of 140 children with NTM disease, comprising 107 cases of lymphadenitis, 25 cases of SSTIs and 8 cases with other types of infections were identified. Demographic details and laboratory results at presentation are shown in Table 1.

**Table 1:** Demographic characteristics, symptoms, and laboratory findings at presentation in the three groups with nontuberculous mycobacterial disease.

	NTM all cases (n = 140)	NTM lymphadenitis (n = 107)	NTM skin & soft tissue infections (n = 25)	NTM other sites of infection(n = 8)	p-value *
Age at presentation, median (IQR)	2.8 (2.1–5.5)	2.6 (2.1–3.8)	8.9 (4.5–12.6)	5.5 (2.2–12.0)	**< 0.0001**
Male gender, no. (%)	66/140 (47.1%)	48/107 (44.9%)	14/25 (56.0%)	4/8 (50.0%)	0.5954
Born overseas, no. (%)	4/105 (3.8%)	3/84 (3.6%)	1/15 (6.7%)	0/6 (0.0%)	0.7465
**Constitutional symptoms**					
Fever, no. (%)	18/126 (14.3%)	12/94 (12.8%)	2/24 (8.3%)	4/8 (50.0%)	**0.0100**
Malaise, no. (%)	12/126 (9.5%)	8/94 (8.5%)	2/24 (8.3%)	2/8 (25.0%)	0.3050
Weight loss, no. (%)	4/126 (3.2%)	2/94 (2.1%)	0/24 (0.0%)	2/8 (25.0%)	**0.0012**
Night sweats, no. (%)	4/126 (3.2%)	3/94 (3.2%)	1/24 (4.2%)	0/8 (0.0%)	0.8440
**Laboratory results**					
WBC, median (IQR)	9.5 (7.8–12.1)	9.6 (7.7–12.2)	8.4 (6.9–10.3)	11.7 (8.8–14.7)	0.0982
Neutrophil count, median (IQR)	4.3 (3.3–6.7)	4.3 (3.0–6.7)	4.3 (3.6–5.6)	3.9 (2.7–9.4)	0.9969
Lymphocyte count, median (IQR)	3.8 (2.3–5.2)	3.8 (2.9–5.6)	3.3 (2.1–4.9)	4.0 (1.7–6.9)	0.3443
CRP, median (IQR)	8.0 (8.0–25.0)	8.0 (8.0–31.0)	8.0 (8.0–12.3)	8.0 (5.5–30.0)	0.3876
ESR, median (IQR)	12.5 (8.3–31.5)	10.0 (7.8–30.5)	12.5 (5.8–39.3)	21.5 (17.8–65.0)	0.2285

* Three-group comparison of continuous variables with Kruskal Wallis test or of categorical variables with chi-square test.

Abbreviations used: IQR = interquartile range; no. = number; NTM = nontuberculous mycobacteria.

**Fig 1:**
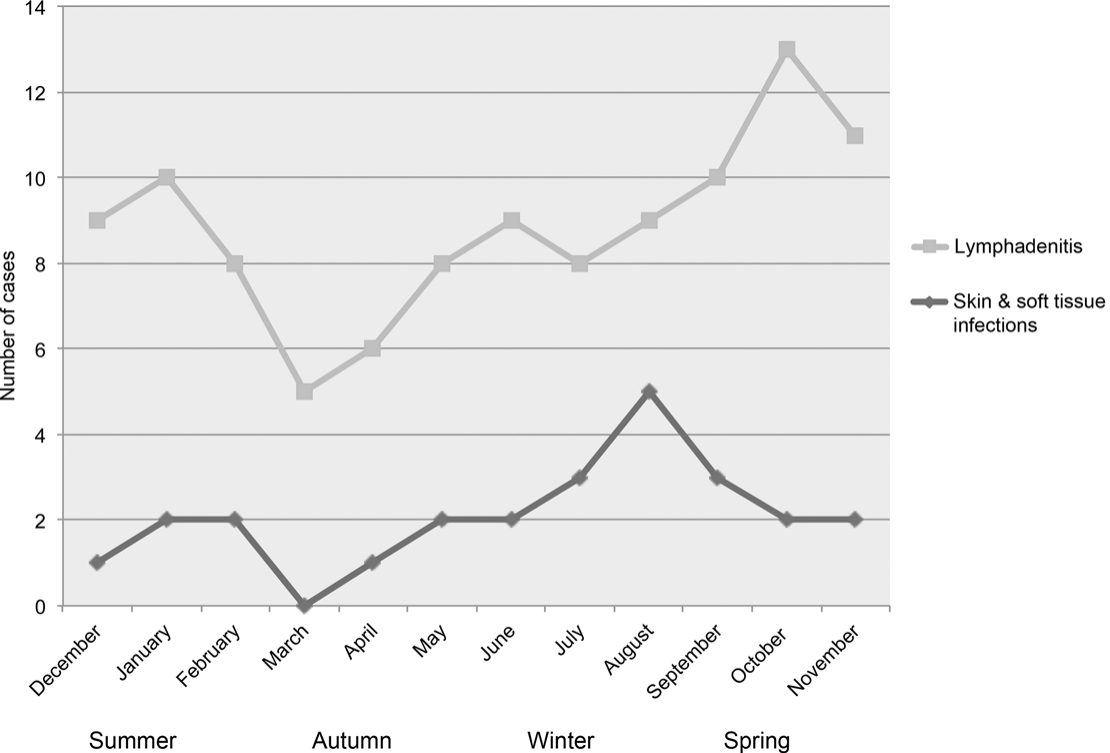
Seasonal distribution of nontuberculous mycobacterial lymphadenitis and skin and soft tissue infections over the study period according to the month of initial presentation.

Annual case numbers varied between 7 per year (in 2009) and 19 per year (in 2004), corresponding to an estimated incidence of NTM disease of 0.6 to 1.6 cases per 100,000 children per year; no increasing trend was observed over the study period. Fig 1 shows the seasonal distribution of cases with NTM lymphadenitis and SSTIs. The highest number of cases occurred in spring and late winter, respectively. In both groups the lowest number of cases occurred around March (autumn in the Southern hemisphere).

**Fig 2:**
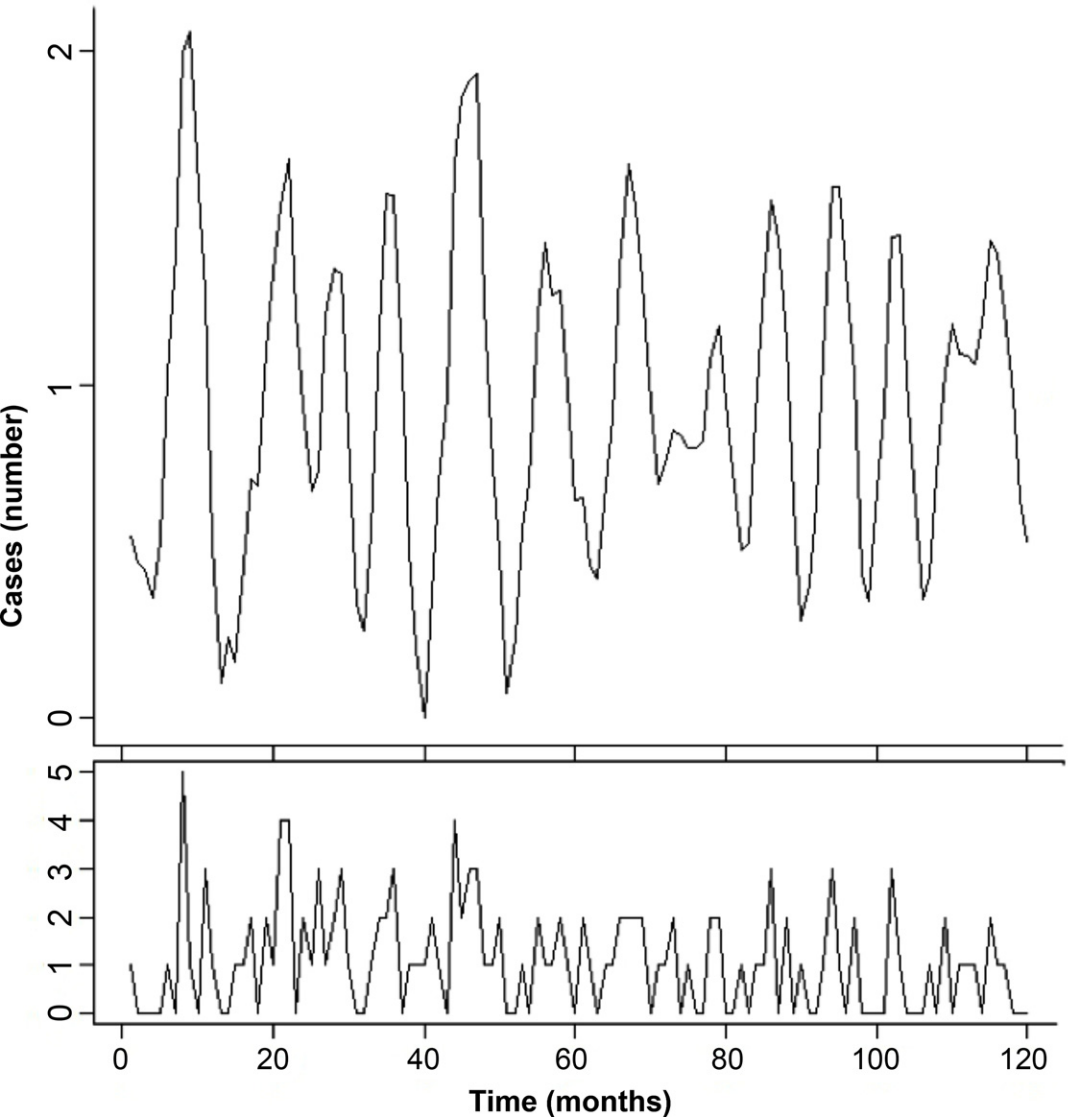
Time series of nontuberculous mycobacterial lymphadenitis and skin and soft tissue infections combined over the study period. The lower plot shows the raw data; the upper plot shows the 3^rd^ level denoised data. Denoising was performed using a discrete wavelet transform with Daubechies length 6 wavelet filter.

**Fig 3:**
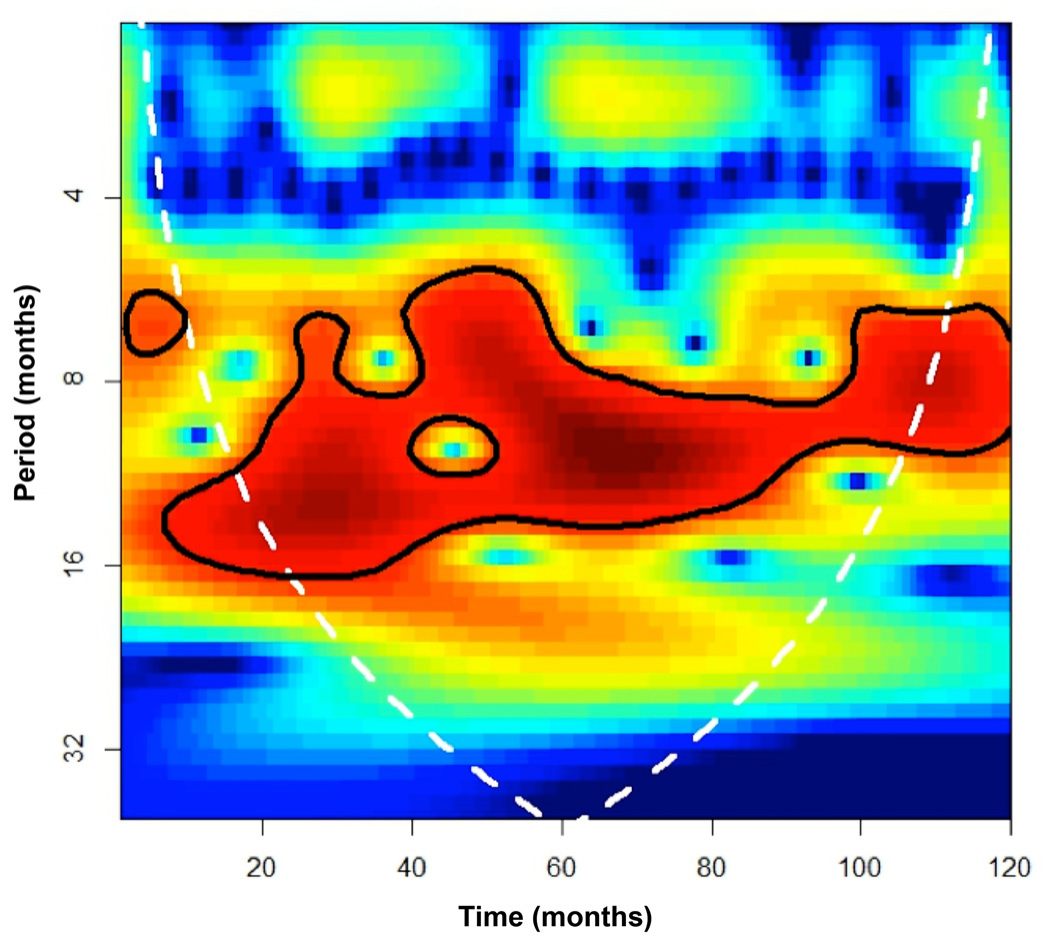
Power spectrum obtained from a continuous wavelet transform based on a Morlet wavelet filter for the detrended and denoised time series of nontuberculous mycobacterial lymphadenitis and skin and soft tissue infection cases combined. The graph shows the power for each period or cycle (y-axis) along the time series (x-axis). High values are shown in dark-red and low values in dark-blue. The dark line contains the statistically significant periods (p<0.05); the white line is the cone of influence that delimits the area not influenced by edge-effects.

The denoising of the time series of NTM lymphadenitis and SSTI cases combined showed the presence of intra-annual and inter-annual cycles (Fig 2). The level with highest peak signal-to-noise ratio was the 3^rd^ denoised level, which contained peaks generally collocated at the end of each calendar year (spring in the Southern hemisphere; Fig 2, upper plot). The subsequent analysis of these cycles using continuous wavelet transform found the occurrence of significant (p<0.05) cycles ranging from 7 to 16 months, with those between 9 and 12 months spanning the majority of the time series (Fig 3).

### NTM lymphadenitis

Among the 107 cases of NTM lymphadenitis, in 92 cases (86.0%) the lymphadenitis affected the submandibular or cervical area, in 8 (7.5%) the preauricular, in 6 (5.6%) the inguinal, and in 1 (0.9%) the axillary area. In the majority of cases, inflammatory parameters were normal or only mildly elevated (Table 1). Local symptoms and signs of infection at presentation comprised erythema in 73.4% (58/79), discoloration of the skin in 15.2% (12/79), swelling in 95.0% (77/81), pain in 20.0% (16/80), tenderness in 30.4% (24/79), fluctuation in 16.7% (13/78), and fistula formation in 2.5% (2/79). Constitutional symptoms at presentation are summarized in Table 1. None of the cases of NTM lymphadenitis had a known underlying immunodeficiency; one had type 1 diabetes mellitus.

The results of investigations for mycobacterial infections are summarized in Table 2. Only few cases underwent a tuberculin skin test (TST) or a QFT assay. Polymerase-chain-reaction (PCR) testing of lymph node material had the highest diagnostic yield (91.3% sensitivity), followed by mycobacterial culture (64.8% sensitivity) and microscopy for acid-fast bacilli (30.3% sensitivity). *Mycobacterium avium* complex (MAC) accounted for all but one of the 62 cases in which a microbiological diagnosis was established at species level (Table 2).

**Table 2:** Results of diagnostic tests for mycobacterial infection, causative species and outcome in the three groups with nontuberculous mycobacterial disease.

	NTM all cases (n = 140)	NTM lymphadenitis (n = 107)	NTM skin & soft tissue infections (n = 25)	NTM other sites of infection (n = 8)	p-value *
**Diagnostic tests**					
Positive TST, no. (%) **	2/11 (18.4%)	1/7 (14.3%)	0/1 (0.0%)	1/3 (33.3%)	0.6850
Positive QFT, no. (%)	1/12 (8.3%)	0/6 (0.0%)	1/2 ^§^ (50.0%)	0/4 (0.0%)	0.0654
Positive stain for acid-fast bacilli,no. (%)	46/129 (35.7%)	30/99 (30.3%)	13/23 (56.5%)	3/7 (42.9%)	0.0562
Positive culture, no. (%)	80/119 (67.2%)	59/91 (64.8%)	13/20 (65.0%)	8/8 (100.0%)	0.1236
Positive PCR, no. (%)	92/100 (92.0%)	63/69 (91.3%)	21/23 (91.3%)	8/8 (100.0%)	0.6852
**Causative species**					
MAC, no. (%)	71 (78.9%)	61 (98.4%)	3 (15.0%)	7 (87.5%)	-
*M*. *ulcerans*, no. (%)	13 (14.4%)	-	13 (65.0%)	-	-
*M*. *marinum*, no. (%)	3 (3.3%)	-	3 (15.0%)	-	-
*M*. *simae*, no. (%)	1 (1.1%)	1 (1.6%)	-	-	-
*M*. *flavescens*, no. (%)	1 (1.1%)	-	1 (5.0%)	-	-
*M*. *terrae*, no. (%)	1 (1.1%)	-	-	1 (12.5%)	-
**Outcome**					
Symptomatic cure at 6 months, no. (%)	122/123 (99.2%)	92/92 (100%)	24/24 (100%)	7/8 (87.5%)	**0.0007**
Recurrence, no. (%)	25/139 (18.0%)	19/107 (17.8%)	4/24 (16.7%)	0/8 (0.0%)	0.4010

* Three-group comparison of continuous variables with Kruskal Wallis test or of categorical variables with chi-square test.

** Positive TST defined as induration of ≥10 mm at 48 to 72 hours.

^§^ The patient with the positive QFT result had *Mycobacterium marinum* infection.

Abbreviations used: IQR = interquartile range; no. = number; NTM = nontuberculous mycobacteria; PCR = polymerase chain reaction; QFT = QuantiFERON-TB Gold assay; TST = tuberculin skin test.

Almost all cases (97.2%; 104/107) underwent complete surgical excision of the affected lymph node or group of lymph nodes. Two cases (1.9%) underwent diagnostic biopsies only; the remaining case (0.9%) had a partial excision. Eight patients (7.5%) developed facial palsy as a complication of surgery; all these cases had submandibular or cervical lymphadenitis. Overall, 14 of the 107 cases received a course of anti-mycobacterial treatment post-operatively (duration ranging from 2–6 months), either with clarithromycin alone (42.9%) or a combination of clarithromycin and rifampicin (57.1%). None of the cases experienced significant adverse events related to anti-mycobacterial treatment; only one reported transient arthralgia. Four of the cases (28.5%) treated with anti-mycobacterial drugs developed one or more recurrences, compared with 15 cases (16.1%) which did not receive anti-mycobacterial treatment (p = 0.2687). Of the 19 cases with recurrence, 16 underwent further surgical intervention. Seven cases had between 2 and 4 more recurrences; none of those cases had received combination treatment with clarithromycin and rifampicin (ie ≥2 recurrences in 0/8 cases (0%) treated with clarithromycin and rifampicin versus 7/99 cases (7.1%) without anti-mycobacterial treatment or clarithromycin only; odds ratio (OR) 0.73; p = 0.6053). In all patients, symptomatic cure was achieved within 6 months of treatment initiation.

### NTM skin and soft tissue infections

Among the 25 cases of NTM SSTIs, the most commonly involved location was a lower limb (52.0%), followed by an upper limb (28.0%), the face (12.0%), the ear (4.0%) and the scalp (4.0%). In the majority of cases inflammatory parameters were normal or only mildly elevated (Table 1). Local symptoms and signs of infection comprised erythema in 69.6% (16/23), discoloration in 30.4% (7/23), pain in 30.4% (7/23), and tenderness in 56.5% (13/23). Eighteen cases (78.2%) had cutaneous ulceration; only one (4.3%) had co-existing regional lymphadenopathy. The constitutional symptoms at presentation are shown in Table 1. Only one of the cases had a known immunodeficiency (isolated IgE deficiency).

The results of investigations for mycobacterial infections are summarized in Table 2. Only few cases had a TST or a QFT assay performed. Notably, one patient with *Mycobacterium marinum* infection had a positive QFT result, as described in an earlier publication [11]. PCR testing of tissue had the highest diagnostic yield (91.3% sensitivity), followed by mycobacterial culture (65.0% sensitivity) and microscopy for acid-fast bacilli (56.5% sensitivity). *Mycobacterium ulcerans* accounted for almost two-thirds of the 20 cases in which a microbiological diagnosis was established at species level (Table 2).

Seventeen cases (68.0%) underwent complete surgical excision, 6 (24.0%) partial excision for diagnostic purposes, and 2 (8.0%) biopsy only. Of the cases which underwent surgical excision, 12 received a course of anti-mycobacterial treatment post-operatively (duration ranging from 1.5–6 months). The most common regimen was a combination of rifampicin and clarithromycin, used in 8 cases; one case each received a combination of rifampicin and erythromycin, rifampicin monotherapy, clarithromycin monotherapy and triple therapy with rifampicin, clarithromycin and ciprofloxacin. All 8 cases which did not undergo surgical excision received anti-mycobacterial treatment (duration ranging from 3–6 months); 7 of those cases received a combination of rifampicin and clarithromycin; one case (with *M*. *marinum* infection) received a combination of rifampicin and ethambutol. None of the cases which received anti-mycobacterial treatment had significant adverse events; two cases had transient diarrhea.

Four cases, all of which had previously undergone surgical excision, had a recurrence; three of these underwent further surgical intervention. Two of these cases had received a regimen comprising two anti-mycobacterial drugs, while one had received clarithromycin only and the remaining one no anti-mycobacterial treatment (ie recurrence in 2/19 cases (10.5%) treated with two or more anti-mycobacterial drugs versus 2/6 (33.3%) treated with one anti-mycobacterial drug or none; OR 0.23; p = 0.2340). All cases were reported to be cured at 6 months after treatment initiation.

### NTM infections affecting other sites

Eight patients had other types of NTM disease, comprising 5 cases with pulmonary infection, and one case each of genitourinary, retropharyngeal and disseminated infection. Demographic details and laboratory results at presentation are shown in Table 1. The results of investigations for mycobacterial infections are summarized in Table 2. Only one case, a patient with pulmonary MAC infection, had a positive TST result.

The cases with pulmonary disease comprised 4 cases with MAC infection and one with *Mycobacterium terrae* infection. Underlying conditions were present in two cases; one had mannose-binding lection deficiency (*M*. *terrae* infection) and one had cystic fibrosis (MAC infection). All five cases had abnormal chest x-rays at presentation. Respiratory symptoms at presentation comprised cough (100%), wheeze (60%), and shortness of breath (40%). The duration of anti-mycobacterial treatment varied between 6 months and 3.5 years. In four cases symptomatic cure was achieved at the end of treatment; the patient with *M*. *terrae* infection showed little improvement after 3.5 years of treatment.

The case with retropharyngeal infection was a 2-year-old, who presented with signs of upper airway obstruction and apneas. A computer-tomography scan revealed retropharyngeal lymphadenopathy. Histology of biopsy tissue showed granulomatous inflammation; MAC was detected by PCR. The patient was treated with rifampicin and erythromycin for 6 months, and made a full recovery.

The case with genitourinary infection was a 5-year-old oncology patient who initially presented with febrile neutropenia. MAC was repeatedly cultured from urine, and PCR tests also confirmed MAC infection. She was successfully treated with a 10-month-course of clarithromycin, ciprofloxacin and ethambutol.

The patient with disseminated MAC infection was a 3-year-old boy with interferon-gamma receptor deficiency, who presented with multifocal osteomyelitis. A biopsy from a rib revealed caseating granulomata on histology; MAC was detected by PCR. He was treated with clarithromycin, rifampicin and ethambutol, and made a full recovery.

## Discussion

To our knowledge this is the largest study of NTM infections in children published to date. In accordance with data from a variety of geographical locations, our findings suggest that NTM disease remains uncommon [1,12–14]. Notably, our estimated incidence of 0.6 to 1.6 cases per 100,000 children per year is remarkably similar to estimates reported by recent studies in Europe [12,13]. Although a few studies have reported a rise in NTM disease in the last two decades, we did not observe an increasing trend [15–17].

We believe our study is the first to describe a seasonal variation in the incidence of NTM disease, with the lowest incidence occurring in autumn and the highest in late winter to spring, with inter-annual cycles typically ranging from 9 to 12 months. This observation is intriguing in light of recent reports of a seasonal variation in the incidence of TB disease [18–20]. In accordance with our findings in NTM disease, most of these studies reported that the incidence of TB peaked in spring, while the lowest incidence was observed in autumn [18,19]. Although the underlying mechanism for this seasonal pattern of TB remains uncertain, it has been postulated that seasonal variation in vitamin D plays a role [18]. Average daily sunshine in Victoria is highest in January and February (ie the months preceding March, when the lowest incidence was observed; average daily sunshine: 8–10 hours), and lowest in June and July (ie the months preceding the rise in case numbers; average daily sunshine: 4–6 hours) [21]. Interestingly, a recent study showed that in the Australian population, 25-hydroxyvitamin D levels are on average lowest in August and September followed by a slow increment over the following months [22], which coincides with the time period when the highest numbers of cases of NTM disease were observed our study. There is considerable evidence supporting the notion that vitamin D plays an important part in the immune response to TB [23]. However, only a few studies have reported an association between vitamin D deficiency or vitamin D receptor gene polymorphism and NTM disease [24,25].

Lymphadenitis accounted for the clear majority of cases of NTM diseases in our study, consistent with studies in other geographical locations [13,14,26,27]. The comparatively high proportion of NTM SSTIs is likely explained by the fact that major Australian epicenters of *M*. *ulcerans* infection extend to within 50 kilometers of our hospital. In Australia, the disease is commonly referred to as ‘Bairnsdale ulcer’ (rather than ‘Buruli ulcer’) based on a report from that area in 1948, which included the first description of the causative organism [28]. Our study population included three cases of *M*. *marinum* infection, despite only a few pediatric cases being reported in the literature [11,29]. This likely relates to the fact that a large proportion of the population served by our hospital lives in close proximity to coastal waters, and the popularity of swimming and other watersports, which are significant risk factors for *M*. *marinum* infection [29].

NTM infections other than lymphadenitis and SSTIs accounted for only a small proportion of cases. MAC was the most frequent cause of pulmonary NTM infection, in keeping with a recent review of pulmonary NTM disease in immunocompetent children [30]. It has been suggested that the incidence of pulmonary NTM disease is rising, particularly in patients with cystic fibrosis [31,32]. Improved survival and selection of NTM through antibiotic pressure are the likely explanation for the increase in this particular patient group [32].

Our study highlights the superiority of molecular methods over conventional culture for the diagnosis of NTM disease. Only few cases had undergone testing with QFT assays, partly because these were only licensed three years into the study period. However, the case with *M*. *marinum* infection with a positive result highlights that a positive QFT assay can also occur in infections with NTM species that express one or more of the stimulatory antigens used in this assay (ie ESAT-6, CFP-10 and TB 7.7), which includes *M*. *marinum*, *M*. *kansasii* and *M*. *szulgai* [11,33].

There remains widespread controversy regarding the optimal management of NTM lymphadenitis [34–36], primarily as a result of limited data. Most previous publications report small cohorts, often treated with a myriad of anti-mycobacterial regimens. To date, only one randomized controlled trial comparing surgical intervention and anti-mycobacterial treatment has been published [37]. In that study, 100 children with cervicofacial NTM lymphadenitis were randomized to surgical excision or conservative therapy with a regimen of clarithromycin and rifabutin. Cure at 6 months (as defined by the authors) was achieved in 96% of the surgical compared with 66% in the conservative treatment group, leading the authors to conclude that surgical treatment was superior. However, a number of aspects complicate the interpretation of the study findings. Firstly, patients underwent diagnostic fine needle aspiration prior to randomization, a procedure associated with fistula formation and poor healing [1,38], which likely disadvantaged the conservative treatment group. Secondly, in that group, three of the NTM isolates were found to be resistant to clarithromycin, and a further three resistant to rifabutin. Finally, patients in the conservative treatment arm who showed no improvement at the 3-month-review were classified as ‘treatment failure’ and subjected to surgery, rather than being continued on anti-mycobacterial therapy; this applied to 10 patients, which contributed significantly to the total of 17 cases of ‘treatment failure’.

Surgical treatment of cervicofacial NTM lymphadenitis is associated with significant risks [35]. Apart from the potential complications associated with general anaesthesia, well-chronicled complications include post-operative wound infections, hematoma formation, and–most importantly–facial palsy. In the aforementioned study, 28% of patients in the surgical treatment arm had surgical complications, which included facial palsy in 14%, with one case (2%) being permanent [37]. Our data compare favorably, with only 7.5% of patients who underwent surgery for NTM lymphadenitis developing facial palsy. However, importantly, our data suggest that in comparison the risks associated with conservative treatment are low, as none of the patients who received anti-mycobacterial treatment experienced significant adverse events.

Our data do not clarify whether conservative treatment alone is a suitable alternative to surgery, as almost all patients with NTM lymphadenitis underwent surgery. We found that the proportion of patients with recurrence appeared to be higher in cases which received anti-mycobacterial treatment (in addition to surgery). However, this is likely explained by the fact that children with extensive disease and/or incomplete excision were more likely to have been started on anti-mycobacterial treatment than those with less extensive disease (ie selection bias). Interestingly, we found that multiple recurrences were less common in children who were treated with a combination of clarithromycin and rifampicin compared with cases which received clarithromycin only or no anti-mycobacterial treatment. These findings indicate that in populations where the majority of NTM lymphadenitis is caused by MAC, adjunctive clarithromycin/rifampicin combination therapy may be beneficial for reducing the risk of multiple recurrences.

The optimal treatment of NTM SSTIs also continues to be debated, again as a result of limited high-quality data. However, a recent randomized trial in patients with *M*. *ulcerans* infection showed that conservative treatment with two anti-mycobacterial drugs is successful in the majority of cases with early limited disease [39]. Many experts recommend the use of a combination of two anti-mycobacterial drugs to treat *M*. *marinum* infections, although the data regarding treatment of this species are less robust [29]. This is in accordance with the latest ATS/IDSA statement, which highlights the limited evidence to support this particular recommendation [6]. Although most of the patients with SSTIs in this study underwent surgery, thereby precluding conclusions regarding the efficacy of conservative treatment alone, we found that the proportion of patients experiencing a recurrence was considerably smaller in the group of cases treated with two anti-mycobacterial drugs compared with those which received only one anti-mycobacterial drug or none.

### Limitations

The main limitation of this study lies in its retrospective nature, which resulted in some missing data, mainly related to presenting symptoms and signs. However, all data relating to time of presentation, treatment and outcome were complete, highlighting the robustness of the main study findings. Our incidence estimates are based on census data from 2006 and the assumption that the paediatric population in Victoria remained stable over the study period. This is supported by official census data (available at: http://www.censusdata.abs.gov.au) showing that between 2006 and 2011 there was only an approximate 5% increase in the number of Victorian residents 0 to 14 years of age. Nevertheless, this approach may have resulted in minor imprecisions in our incidence estimates. Although our hospital is the largest paediatric referral center in Victoria by far, it is possible that a small number of cases with NTM disease received care elsewhere. Therefore, the true incidence of NTM disease may be higher than our estimates. One further potential limitation is that the seasonal data are based on the time of presentation to specialist services in our hospital, rather than the onset of the disease. However, the threshold for referrals for a specialist opinion in our setting is low and waiting times are relatively short; therefore significant delays between disease onset and assessment in the hospital are uncommon.
